# TMT-based quantitative proteomics analysis of the effects of Jiawei Danshen decoction myocardial ischemia-reperfusion injury

**DOI:** 10.1186/s12953-022-00200-7

**Published:** 2022-12-14

**Authors:** Xiang-Mei Zhu, Yang Tan, Yu-He Shi, Qing Li, Jue Zhu, Xiang-Dan Liu, Qiao-Zhen Tong

**Affiliations:** 1 Pharmacy of College, Hunan University of Chinese Medicine, Xueshi Road, Number 300, Changsha, Hunan 410208, People’s Republic of China; 2 The Second Hospital of Hunan University of Chinese Medicine, Caie North Road, Number 233, Changsha, Hunan 410005, People’s Republic of China; 3 Key Laboratory of Germplasm Resources and Standardized Planting of Bulk Authentic Medicinal Materials from Hunan, Xueshi Road, Number 300, Changsha, Hunan 410208, People’s Republic of China

**Keywords:** Myocardial ischemia-reperfusion injury (MIRI), Tandem mass tag (TMT), Jiawei Danshen decoction (JWDSD), Proteomic, Traditional Chinese medicine (TCM)

## Abstract

**Background:**

Every year, approximately 17 million people worldwide die due to coronary heart disease, with China ranking second in terms of the death toll. Myocardial ischemia-reperfusion injury (MIRI) significantly influences cardiac function and prognosis in cardiac surgery patients. Jiawei Danshen Decoction (JWDSD) is a traditional Chinese herbal prescription that has been used clinically for many years in China to treat MIRI. The underlying molecular mechanisms, however, remain unknown. To investigate the proteomic changes in myocardial tissue of rats given JWDSD for MIRI therapy-based proteomics.

**Methods:**

MIRI rat model was created by ligating/releasing the left anterior descending coronary artery. For seven days, the drugs were administered twice daily. The model was created following the last drug administration. JWDSD's efficacy in improving MIRI was evaluated using biochemical markers and cardiac histology. Tandem mass tag-based quantitative proteomics (TMT) technology was also used to detect proteins in the extracted heart tissue. To analyze differentially expressed proteins (DEPs), bioinformatics analysis, including gene ontology (GO) and Kyoto encyclopedia of genes and genomes (KEGG) pathways, were employed. Furthermore, western blotting confirmed the potential targets regulated by JWDSD.

**Results:**

The histopathologic characteristics and biochemical data showed JWDSD's protective effects on MIRI rats. A total of 4549 proteins were identified with FDR (false discovery rate) ≤1%. Twenty overlapping were identified (162 DEPs and 45 DEPs in Model/Control or JWDSD/Model group, respectively). Of these DEPs, 16 were regulated by JWDSD. GO analysis provided a summary of the deregulated protein expression in the categories of biological process (BP), cell component (CC), and molecular function (MF). KEGG enrichment analysis revealed that the signaling pathways of neutrophil extracellular trap formation, RNA polymerase, serotonergic synapse, and linoleic acid metabolism are all closely related to JWDSD effects in MIRI rats. Furthermore, T-cell lymphoma invasion and metastasis 1 (TIAM1) was validated using western blotting, and the results were consistent with proteomics data.

**Conclusions:**

Our study suggests that JWDSD may exert therapeutic effects through multi-pathways regulation in MIRI treatment. This work may provide proteomics clues for continuing research on JWDSD in treating MIRI.

**Supplementary Information:**

The online version contains supplementary material available at 10.1186/s12953-022-00200-7.

## Introduction

Myocardial ischemia is a severe risk to patients suffering from cardiovascular disease [1]. Myocardial ischemia-reperfusion injury (MIRI) is defined as tissue damage that occurs when early and rapid coronary flow returns to the heart after ischemia, frequently exacerbating the myocardial injury [2]. Four different forms of myocardial reperfusion injury are recognized: lethal arrhythmias, lethal myocardial reperfusion injury, myocardial stunning, and microvascular obstruction [3]. MIRI is a significant contributor to the morbidity and mortality of coronary artery disease [4]. MIRI pathogenesis is complex, with oxidative stress, ion accumulation, mitochondrial membrane potential dissipation, endothelial dysfunction, immune response, and other contributing factors. However, the molecular mechanism underlying MIRI remains unknown [5]. β-blockers, adenosine receptor agonists, nitrates and nitroglycerin, mPTP inhibitors, and Na^+^/H^+^ exchange inhibitors are currently widely used to treat heart failure. Still, the efficacy of single-target drugs is limited and challenging to meet clinical needs [6]. Therefore, it is of urgent demand to explore the molecular mechanisms of MIRI and to develop better therapies for preventing and treating MIRI. Traditional Chinese medicine (TCM) has the characteristics of "multi components, multi targets, and multi pathways," and searching for active ingredients from traditional Chinese medicine that protect against myocardial ischemia-reperfusion injury has become a hot research topic both at home and abroad [7, 8]. Jiawei Danshen decoction (JWDSD) evolved from Danshen Yin (DSY) from Shi Fang Ge Kuo, which promotes nourishing Qi, activates blood, and resolves stasis in Chinese medicine theory. As a classic prescription, it has been treating coronary heart disease and angina since its establishment [9]. Table 1 contains a list of seven plant materials. The main components of JWDSD, such as Radix et *Rhizoma salviae* and *Lignum santalialbi*, have been used in Traditional Chinese Medicine (TCM) in China and many Asian countries as preventive or therapeutic remedies for coronary heart disease, vascular diseases, stroke, and hyperlipidemia [10], and our previous experiments also demonstrated JWDSD had an anti-apoptotic effect, anti-oxidative stress effect, anti-inflammatory activities, and mitochondrial protection [11–13]. Nevertheless, the mechanism of its protective effect remains not fully understood.

**Table 1:** Different components in the formula of JWDSD

Chinese name	English name	Botanical name	Species name	Family	Part used	Ratio
Dan Shen	root of red-rooted salvia	Salvia miltiorrhiza Bge	Radix et Rhizoma salviae miltiorrhizae	Lamiaceae	Rhizome and Root	20
Tan Xiang	sandalwood	Santalum album L	Lignum santalialbi	Santalaceae	Heartwood	6
Chuan Xiong	Ligusticum wallichii	Ligusticum chuanxiong Hort	Rhizoma chuanxiong	Umbelliferae	Rhizome	6
Dang Gui	Angelica sinensis	Angelica sinensis (Oliv.) Diels	Radix angelicae sinensis	Umbelliferae	Root	6
Hong Hua	safflower	Carthamus tinctorius L	Flos carthami	Compositae	Flos	6
Chi Shao	radix paeoniae rubrathe root of common peony	Paeonia veitchii Lynch	Radix paeoniae rubra	Paeoniaceae	Root	10
Sheng Di Huang	Glutinous rehmannia	Rehmannia glutinosa (Gaertn.) Libosch. ex Fisch. et Mey	Radix rehmanniae	Scrophulariaceae	Root tuber	12

With the recent advanced development of proteomics technology, the global protein landscape of various samples can be identified within a single experiment, establishing proteomics as a powerful approach for elucidating the underlying mechanisms of TCM [14]. Tandem mass tag-based (TMT) is one of the relative quantitative proteomics technologies, which simultaneously labels and analyzes multiple biological samples, further outputs precise sensitivity and high-quality data [15]. A quantitative TMT-labeled proteomic approach was used to elucidate DEPs between JWDSD group and MIRI model group to elucidate the therapeutic mechanism of JWDSD at the protein level. Furthermore, a bioinformatics analysis of DEPs in the enriched pathways was carried out.

## Materials and methods

### JWDSD preparation

The herbs used in the JWDSD were purchased from the First Affiliated Hospital of Hunan University of Chinese Medicine (Changsha, China) and were composed of Dan Shen (batch number, 20201125), Tan Xiang (batch number, 20201031), Chuan Xiong (batch number, 20201129), Dang Gui (batch number, 20201121), Hong Hua (batch number, 20201110), Chi Shao (batch number, 20201019), and Sheng Di Huang (batch number, 20201127). The botanical raw materials were crushed into pieces and mixed in the ratio of 10:3:3:3:3:5:4(w/w) dissolved in 10×8× total drug weight water, and heated to reflux for 1 h and combined with the drug solution, concentrated to 2 g/ml and stored at 4°C for later use. Furthermore, our laboratory has established JWDSD's strict quality control method [16, 17].

### Research animals and experimental design

Male Sprague-Dawley rats weighing 160~180 g were obtained from the Hunan University of Chinese Medicine Animal Experiment Center (Changsha, China License number: SCXK (Hunan) 2019-0004). All rats were fed at 22±3 °C, 50%±5% relative humidity, and 12 h light/dark cycle. Food and drinking were available ad libitum for one week. The animal experiments were performed with the approval of the Animal Ethics Committee of the Hunan University of Chinese Medicine. They were carried out concerning the Chinese Guide for the Care and Use of Laboratory Animals.

All animals were handled with humane care throughout the experiment. The rats were randomly divided into seven groups (*n*=10): the control group, the model group, the sham-operated group (Sham: normal saline, 1 mL/100g), Diltiazem Hydrochloride Tablets group (Diltiazem-H: 4.32mg/kg), and three groups which fed JWDSD in a low, moderate, or high dosage (JWDSD-D, JWDSD-Z, JWDSD-H: 12.10g, 6.05g and 3.03g/kg, botanical raw materials extracted by distilled water). Each group received continuous gavage twice a day for seven days before modeling. The rats were anesthetized with chloral hydrate (300 mg/kg, intraperitoneal injection) 1 h after the last gavage. Their limbs and heads were immobilized, and a ventilator was inserted (initial frequency 60 times/min, 90 times/min after thoracotomy). To expose the heart, the thoracic cavity is opened, a 6-0 suture needle with a thread about 2 mm is inserted below the left atrial appendage, and the left anterior descending (LAD) coronary artery is ligated [18]. The rats were subjected to LAD coronary artery ligation for 30 min and then to reperfusion for 30 min after the same surgical operation. The sham operation group was threaded but not ligated. At the end of the reperfusion period, blood samples were drawn from the abdominal aorta to separate serum for further analysis by centrifugation at 3000 g for 15 min, and cardiac tissues were collected from each group and stored in a -80 °C refrigerator for later use.

### Creatine kinase and lactate dehydrogenase assays

The blood of rats in each group was centrifuged at 3000 R / min for 20 min, and the upper serum was taken. The Chemray 240 automatic biochemical analyzer was used to measure creatine kinase (CK) and lactate dehydrogenase (LDH) levels in serum.

### Heart histological examination

The fresh cardiac tissue was fixed with 4% paraformaldehyde for more than 24 h, dehydrated with different ethanol concentrations (75%, 85%, 90%, 95%, and 100%), and then immersed in paraffin for embedding. After the wax solidifies, they were cut into slices with a thickness of 4 μm. After being immersed in xylene and ethanol, the paraffin sections were stained with H&E (hematoxylin-eosin, servicebio, China) to observe any histopathological changes.

### TMT -labeled quantitative proteomics

#### Protein extraction

The cardiac tissue samples from the Control group, Model group, and JWDSD-H group were ground into a powder with liquid nitrogen and were lysed with SDT lysis buffer (4% (w/v) SDS, 100 mM Tris/HCl pH 7.6, 0.1 M DTT). The lysate was homogenized by MP homogenizer (24×2, 6.0 M/S, 60 s, twice), and the mixture was homogenized on ice for 20 min, then sonicated three times. Later the mixture was centrifuged at 14000 g for 15 min to get the supernatant. All samples were quantified using the BCA method, and aliquots were stored at -80 °C for further use.

#### Protein digestion, TMT labeling and RP classification

Proteins from each sample were processed by Filter aided proteome preparation (FASP) method [19] was used for trypsin digestion, the filtrate was collected, and the peptide was quantified (OD280). Tandem mass tags TMT10 (Thermo Fisher Scientific, USA) with varying molecular weights (126–131 Da) were used as isobaric tags for relative and absolute quantification. According to manufacturer's protocols, the digested samples were individually labeled with TMT10 reagents for 1 h as follows: 100 μg of aliquots of digested peptides of the Control group, Model group, or JWDSD-H group were each labeled with a different isobaric tag (TMT126, 127, 128, 129, 130 and 131, respectively). The labeling reaction was stopped with 5% hydroxylamine. As directed, the Pierce high pH reversed-phase fractionation kit (Thermo scientific) was then used to fractionate TMT-labeled digest samples into 10 fractions using an increasing acetonitrile step-gradient elution.

#### LC-MS/MS analysis

Each fraction was injected for nano LC-MS/MS analysis. The peptide mixture was loaded onto a reverse-phase trap column (Thermo Scientific Acclaim PepMap100, 100μm*2cm, nanoViper C18) connected to the C18 reversed-phase analytical column (Thermo Scientific Easy Column, 10 cm long, 75 μm inner diameter, 3 μm resin) in buffer A (0.1% Formic acid) and separated with a linear gradient of buffer B (84% acetonitrile and 0.1% Formic acid) at a flow rate of 300 nl/min controlled by IntelliFlow technology. Subsequently, peptides were eluted over 90 min using the following gradient: 0-55% buffer B for 80 min, 55-100% buffer B for 5 min, and held in 100% buffer B for 5 min.

The peptides were separated using an HPLC system, then injected into a capillary ion source for ionization and analyzed using Q-Exactive (Thermo Scientific) mass spectrometry. In positive ion mode, the mass spectrometer was used. MS data for HCD fragmentation were collected using a data-dependent top 10 method that dynamically selected the most abundant precursor ions from the survey scan (300-1800 m/z). The automatic gain control (AGC) target was set to 3e6, and the maximum injection time to 10 ms. The dynamic exclusion duration was 40.0 s.

#### Database search

MS/MS spectra were searched using MASCOT engine (Matrix Science, London, UK; version 2.2) embedded into Proteome Discoverer 1.4. The parameters were set as follows: specific enzyme was trypsin; Peptide Mass Tolerance was ±20 ppm; Fragment Mass Tolerance was 0.1Da; Peptide FDR≤0.01; the protein ratios are calculated as the median of unique peptides of proteins. The other parameters were default. DEPs were satisfied following conditions: unique peptides ≥ 2 with average ratio-fold change > 1.2 (up-regulation) and < 0.83 (down-regulation), as well as *p*-value < 0.05.

#### Bioinformatics analysis

The protein sequences of DEPs were in batches retrieved from the UniProtKB database (Release 2016_10) in FASTA format. The term gene ontology (GO) (http: //geneontology. org/) was used to describe cellular components (CC), elucidate biological process (BP), and molecular function (MF). KAAS (http: //www.genome. jp/kaas-bin/kaas_main) was applied to annotate the description of DEPs in KEGG database. KEGG pathway enrichment analysis was performed using Fisher’s exact test.

### Western blot analysis

Protein concentrations were determined using a BCA Protein Concentration Determination Kit after extracting proteins from approximately 100 mg of cardiac tissue (Beyotime). A 40 g protein sample was extracted, separated by SDS-PAGE gel, transferred to PVDF membrane, and blocked with 3% BSA-TBST for ten minutes at 28 °C. The membranes were then incubated overnight at 4 °C with corresponding primary antibodies. Santa Cruz Biotechnology provided TIAM1, GAPDH genes (Dallas, TX, USA. After washing three times (5 min each time) using TBST, the membranes were incubated with secondary antibody for 60 min at room temperature. ECL solution was added to regulate the exposure conditions, and the optical density value of the target zone was analyzed using ImageJ software processing system.

### Statistical analysis

IBM SPSS Statistics V21.0 was used to analyze data, expressed as mean±standard deviation (SD). Independent t-tests or one-way analyses of variance were used to make comparisons. At *P* < 0.05, differences were considered statistically significant.

## Results

### Effects of JWDSD on MIRI rats

**Fig. 1:**
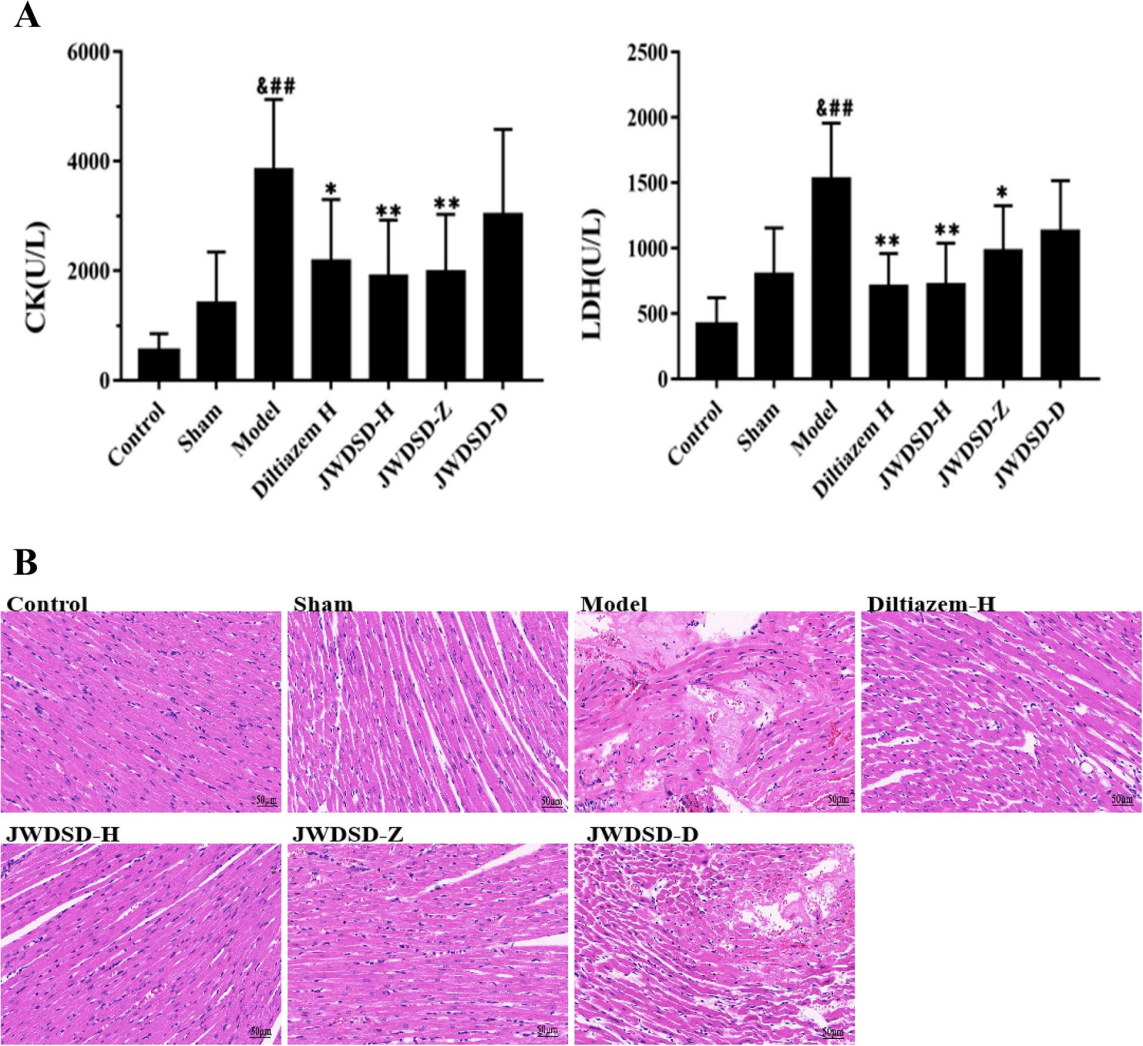
JWDSD pharmacodynamic evaluation (*n*=6, scale bar=50 μm, data are presented as mean±SD. ^##^*P* < 0.01, versus Control group; &*P*<0.01, versus Sham group; ^*^P < 0.05,^**^P < 0.01, versus Model group). **A** Concentration analysis of serum creatine kinase and serum lactic dehydrogenase. **B** Effects of JWDSD on histopathological examination by H&E. Control: control group, Model: MIRI model group, JWDSD-H: high-dose JWDSD+MIRI model group, JWDSD-Z: medium-dose JWDSD+MIRI model group, JWDSD-D: low-dose JWDSD+MIRI model group, Diltiazem-H: diltiazem hydrochloride+MIRI model group, Sham: Sham-operated group

To evaluate whether JWDSD protects myocardium injured by ischemia-reperfusion, the levels of serum CK and LDH were monitored in this study. Compared to the Control group, the MIRI model group showed a marked increase in CK, and LDH levels, while the JWDSD-H significantly decreased CK and LDH compared to the MIRI model group (Fig. 1A). In H&E (Fig. 1B), In the Model group, many myocardial fibers were necrotic and ruptured, with more erythrocyte exudation, scattered nuclei, and many inflammatory cells infiltrated. Concurrently, the above phenomena were significantly reduced in the JWDSD-H pretreatment group, consistent with the results displayed in Fig. 1A. JWDSD can lower the levels of myocardial enzymes CK and LDH and improve the pathological morphology of myocardial tissue, thereby protecting the damaged myocardium.

### TMT quantitative of DEPs

**Fig. 2:**
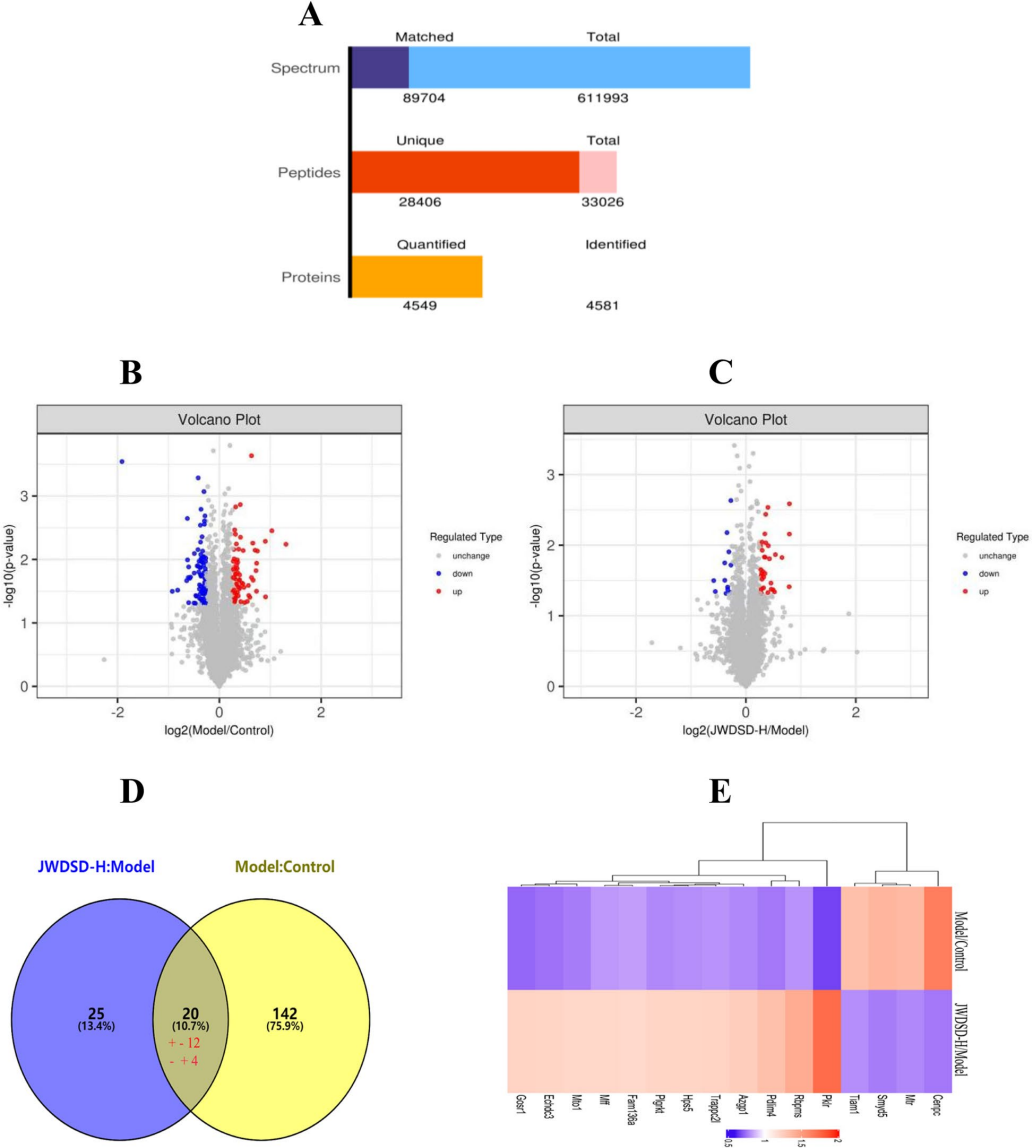
Identification and quantitative analysis of proteins. **A** The total identified proteins in MS analysis. The volcano plot showed the up-(red) or downregulated (blue) proteins between Model/Control groups (**B**) and JWDSD-H/Model groups (**C**) of 4549 proteins. **D** Venn diagram of different expression proteins and their overlap, the "+" indicates up-regulated proteins, while the "-" indicates down-regulated proteins, the "+ -" indicates proteins up-regulated in JWDSD-H/Model groups but down-regulated in Model/Control groups, the "- +" indicates proteins down-regulated in JWDSD-H/Model groups but up-regulated in Model/Control groups. Thresholds: up-regulated fold change ≥1.2, down-regulated fold change ≤ 0.83, Student t-test *p*-value < 0.05. **E** Hierarchical cluster analysis of 16 different expression proteins, the color indicates fold changes of proteins; deep purple indicates an increase while orange indicates a decrease.

The outcomes were compared among the three groups (Control, Model and JWDSD-H). Three replicated experiments were carried out for each comparison group. Through spectral analysis, 33,026 peptides were identified, with 4581.0 proteins identified, 4549.0 of which could be quantified (Fig. 2A). The criteria mentioned above were used to screen DEPs out of 4549.0 quantifiable proteins. In Fig. 2B and C, volcano plots depict the relative changes in protein levels. Moreover, 4501.0, 4500.0 and 4500.0 proteins were quantified in the Model/Control, JWDSD-H/Model, and JWDSD-H/Control, respectively. In the Model/Control group, 162 DEPs were identified (72 were up-regulated and 90 were down-regulated). In the JWDSD-H/Model group, 45 DEPs were appraised (33 were up-regulated and 12 were down-regulated), respectively (Table 2). It is clear that 20 overlapping DEPs were found in both the Model/Control and the JWDSD-H/Model groups (Fig. 2D). JWDSD treatment reversely regulated 16 proteins out of the 20 DEPs. Table 3 and Fig. 2E provide information on 16 DEPs.

**Table 2:** The quantity of DEPs identified in experiments

Sample pairs	Model/Control	JWDSD-H/Model	JWDSD-H/Control
Quantified	4501	4500	4500
Up-regulated	72	33	34
Down-regulated	90	12	25
Total difference	162	45	59

**Table 3:** 16 different expression proteins regulated by JWDSD

Majority protein ID	Gene name	Protein description	Sequence Coverage	Unique peptides	*P* value	Fc (Model/Control)	Fc (JWDSD-H/Model)
E9PSW8	Cenpc	Centromere protein C	1.75	1	0.01	1.65	0.76
D3ZII8	Smyd5	SMYD family member 5	4.32	2	0.02	1.38	0.77
D3ZWV8	Tiam1	T-cell lymphoma invasion and metastasis 1	0.44	1	0.004	1.33	0.80
B0BN94	Fam136a	Protein FAM136A	5.80	1	0.04	0.83	1.20
A0A0G2KAL9	Mff	Mitochondrial fission factor	16.84	4	0.01	0.82	1.21
B2RYU6	Trappc2l	Trafficking protein particle complex subunit 2-like	6.47	1	0.01	0.81	1.23
F2Z3S5	Rbpms	RNA-binding protein, mRNA-processing factor	17.39	3	0.01	0.81	1.43
F1LS35	Hps5	Hermansky-Pudlak syndrome 5 protein homolog	6.15	1	0.04	0.80	1.22
D4ACN8	Plgrkt	Plasminogen receptor (KT)	8.84	2	0.01	0.79	1.22
Q3B8R6	Azgp1	Alpha-2-glycoprotein 1, zinc	2.36	1	0.01	0.79	1.26
F1LYH3	Mto1	Mitochondrial tRNA translation optimization 1	1.36	1	0.02	0.77	1.21
M0R4H5	Pdlim4	PDZ and LIM domain protein 4	2.73	1	0.01	0.76	1.33
Q3MIE0	Echdc3	EnoylCoA hydratase domain containing 3	12.25	2	0.01	0.75	1.22
Q62931	Gosr1	Golgi SNAP receptor complex member 1	3.60	1	0.03	0.73	1.23
A0A0H2UI07	Pklr	Pyruvate kinase L/R	3.58	2	0.01	0.67	1.73
Q9Z2Q4	Mtr	Methionine synthase	0.72	1	0.02	1.36	0.79

### Bioinformatics analysis of DEPs and JWDSD

**Fig. 3:**
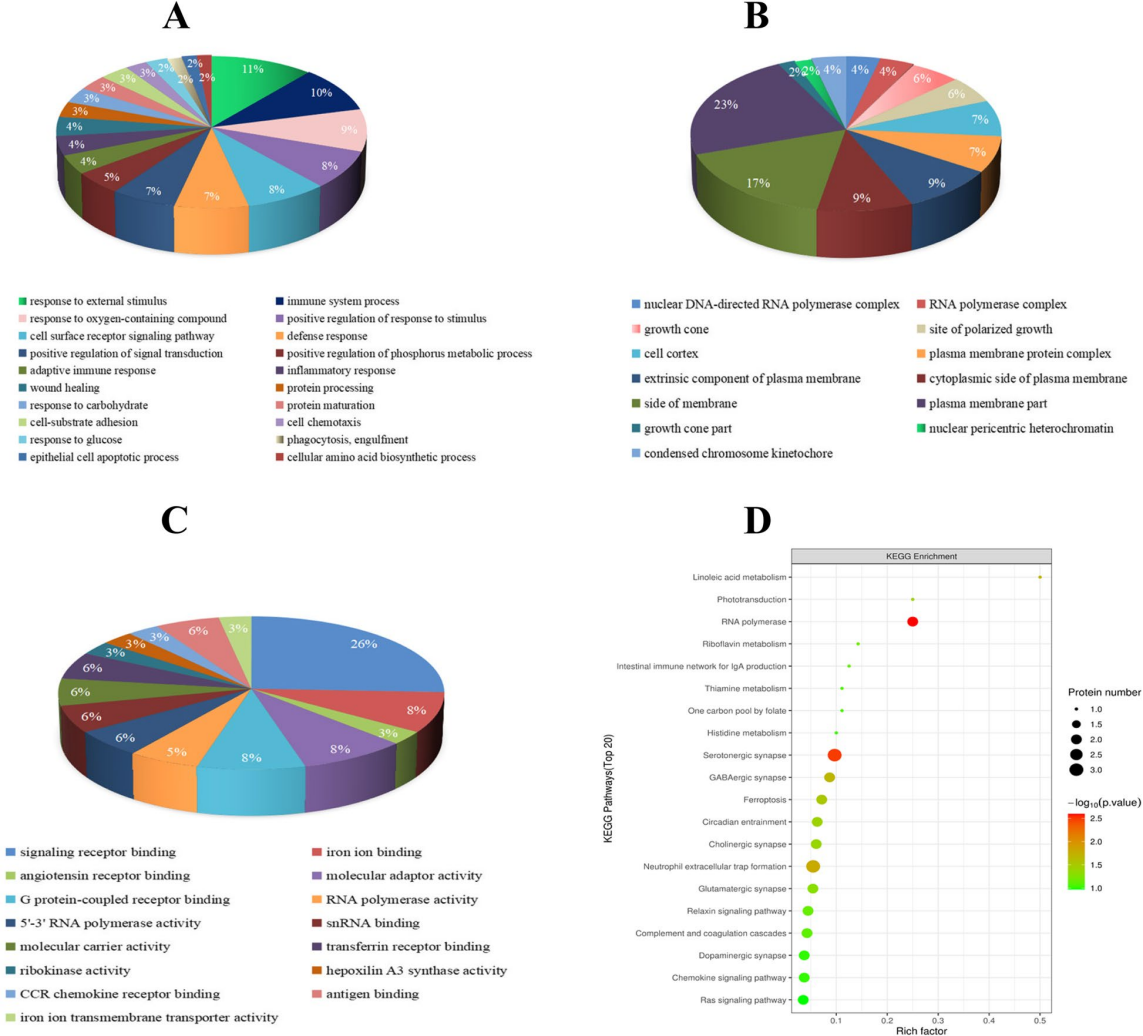
Bioinformatics analysis of DEPs. Pie chart depicting DEPs characterized by GO category, GO annotation of biological processes (**A**), cellular component (**B**) and molecular function (**C**). **D** KEGG pathway analysis between Model and JWDSD-H groups.

To investigate the biological role of DEPs screened to prevent and treat MIRI in JWDSD. GO annotation classification analysis was performed to evaluate the functional annotations of DEPs, and the results were revealed in the biological process. DEPs mainly participated in response to external stimulus (11%), immune system process (10%), response to oxygen-containing compound (9%), positive regulation of response to stimulus (8%), cell surface receptor signaling pathway (8%), defense response (7%) (Fig. 3A). About cellular component, the proteins focused on plasma membrane part (23%), side of membrane (17%), cytoplasmic side of plasma membrane (9%), extrinsic component of plasma membrane (9%), and cell cortex (7%) (Fig. 3B). In molecular function, the main were signaling receptor binding (26%), ion binding (8%), molecular adaptor activity (8%), transferrin receptor binding (6%), and molecular carrier activity (6%) (Fig. 3C). Furthermore, we performed KEGG pathway enrichment analysis and GO annotation enrichment analysis to gain insight into the functions of 45 DEPs. The top 20 enriched pathways or annotations are displayed in Fig. 3, 6 KEGG pathways were filtered out as significant enriched pathways (*P*<0.05); the enriched pathways were serotonergic synapse, neutrophil extracellular trap formation, Linoleic acid metabolism, ferroptosis, RNA polymerase, and cholinergic synapse signaling pathway (Fig. 3D).

### Western blotting validation of TIAM1 protein expression in rat tissue

**Fig. 4:**
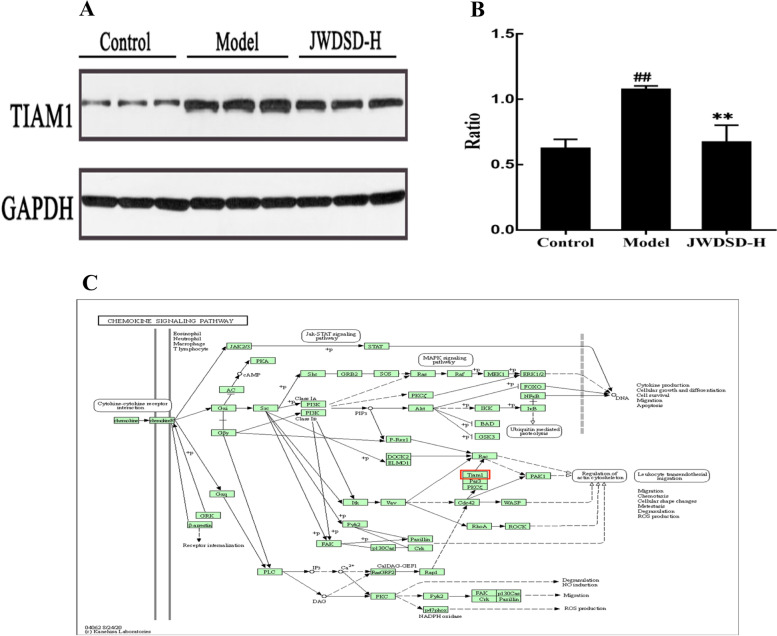
Effects of JWDSD on TIAM1 protein expression in MIRI rats. **A** Western blot. **B** Gray-level scores. **C** TIAM1 participates in the chemokine signaling pathway. Control: control group, Model: MIRI model group, JWDSD-H: high-dose JWDSD+MIRI model group. Values are expressed as mean±SD in each group. ^##^*p*<0.01, versus Control group,^**^P<0.01, versus Model group.

Western blotting was used to detect the expressions of T-cell lymphoma invasion and metastasis 1 (TIAM1) to validate DEPs from the quantitative proteomics research. The results revealed that TIAM1 expression was up-regulated in the Model group but down-regulated in JWDSD-H group (Fig. 4A and B). It participated in chemokine signaling pathway (Fig. 4C). These findings agreed with the findings of the proteomic data.

## Discussion

Previous research found that pretreatment with Danshen ethanol extract can reduce oxidative injury in rats subjected to myocardial ischemia-reperfusion and salvianolic acid B in Danshen can protect against myocardial ischemic injury by promoting mitophagy [20, 21]. Tanxiang can protect cardiac tissue from oxidative stress-induced cell injury and lipid peroxidation [22]. Studies have revealed the modulation of cardioprotection of Chuanxiong in myocardial ischemia injury through the activation of PI3K/Akt/mTOR signaling pathway [23]. Danggui has been revealed to significantly reduce Angiotensin II-induced apoptosis in cardiomyoblast cells, which is mediated by JNK and PI3k inhibitors [24]. Previous studies have demonstrated that total paeony glycosides in Chishao have protective effects on myocardial ischemia induced by isoprenaline and can prevent oxidative damage in ischemia by scavenging free radicals [25]. Dihuang extract can enhance the mobilization, migration, and therapeutic angiogenesis of EPCs after MI by activating SDF-1a/CXCR4 cascade [26]. Furthermore, Hydroxysafflor Yellow A in Honghua has been shown to reduce myocardial injury in vitro and in vivo by inhibiting oxidative stress and apoptosis, with JAK2/STAT1 signaling pathway playing a role [27]. Therefore, previous findings suggest that Danshen, Tanxiang, Chuanxiong, Danggui, Chishao, Dihuang, and Honghua can effectively reduce myocardial injury.

The findings of our experimental study are summarized below. First, we used serum biochemical indexes and cardiac pathology to confirm the cardioprotective function of JWDSD pretreatment in MIRI model rats. Second, using LC-MS/MS, we systematically identified a list of DEPs in JWDSD-treated MIRI rats. We found 20 overlapping DEPs JWDSD-H/Model group and Model/Control group, including 16 proteins that were reversely regulated by JWDSD treatment—AZGP1, ECHDC3, FAM136A, PKLR, GOSR1, HPS5, CENPC, SMYD5, TIAM1, MFF, MTO1, TRAPPC2L, MTR, PLGRKT, RBPMS, and PDLIM4—that served as candidates target to explain the cardioprotective effect of JWDSD. Third, the bioinformatic analysis on DEPs jointly demonstrated that up-regulated proteins of JWDSD-H/Model were mainly concentrated in neutrophil extracellular trap formation, RNA polymerase, serotonergic synapse, and linoleic acid metabolism signaling pathway. In contrast, the down-regulated proteins were primarily focused on Ferroptosis, the cholinergic synapse signaling pathway. Finally, western blotting demonstrated that JWDSD effectively counteracted MIRI-induced TIAM1 downregulation. This study provided proteomics evidence for JWDSD pretreatment of MIRI.

From the differential proteins shared by the three groups, 16 proteins with significant differences were preliminarily screened. TIAM1 was up-regulated in the Model/Control comparison group of proteomics experiment but down-regulated in the JWDSD-H/Control comparison group and involved chemokines signaling pathway. A literature review revealed that chemokines could activate inflammation-related signaling pathways, and MIRI is closely related to the inflammatory response. Therefore, in this experiment, TIAM1 was selected for proteomic validation. TIAM1 is 1591 amino acids long with a molecular weight of 177 kD [28]. TIAM1 serves a critical role in regulating cell adhesion, invasion, and migration [29]. In addition, TIAM1 is a RAC guanine nucleoside exchange factor (GEF) that activates Rac1 [30].

Rac1 has a vital role in cell motility and adhesion, cell proliferation, cell differentiation and apoptosis, and immune regulation. Studies have revealed that IL-8 can increase endothelial cell migration via PI3K/Rac1/RhoA signaling [31]. Early research found that TIAM1 is a specific guanine nucleotide exchange factor for Rac1 and is crucial for cell-cell adhesion and migration. It has also been shown to play a pivotal role in cardiac hypertrophy associated with heart failure [32]. Furthermore, TIAM1 is involved in the chemokine signaling pathway. Chemokines and inflammatory factors are the most direct cause of MIRI, causing inflammatory cell migration and cardiomyocyte death by activating the inflammatory signal pathway [33, 34]. The activation and migration of various inflammatory cells into the vessel wall are regulated by chemokines and chemokine receptors [35]. Tanshinone IIA exerted its cardioprotective effect by lowering MCP-1 expression and macrophage infiltration, thereby dampening inflammatory responses after myocardial infarction [36]. Tetramethylpyrazine exerts potent effects in inhibiting neovascularization, fibrosis, and thrombosis under pathological conditions, and the underlying mechanism may be related to the down-regulation of CXCR4 expression [37]. In addition, Targeting Rac1 signaling has been demonstrated to exert anti-inflammatory effects in models of reperfusion injury, endotoxemia, and acute pancreatitis [38], and TIAM1 affects cell migration by activating Rac1, Tanshinone IIA, and Tetramethylpyrazine are the main chemical components of Jiawei Danshen Decoction. The western blot verification results of this study demonstrated that, when compared to the Model group, the JWDSD-H group down-regulated the expression level of TIAM1 in the myocardial tissue of MIRI rats, indicating that JWDSD reduced the migration of inflammatory cells to the damaged myocardial tissue, reduced the occurrence of inflammatory response, and relieved the myocardial tissue. The damage could be linked to a decrease in TIAM1 expression in rat myocardial tissue.

This is the first study to use LC-MS/MS quantitative proteomics to identify DEPs in MIRI treated with JWDSD. The findings confirmed that JWDSD has a distinct protein profile that suggests adaptive mechanisms in acute MIRI. However, some unknown proteins or proteins outside the detected proteins may be overlooked, as our study only focuses on protein level regulation. Therefore, more molecular biology experiments are required for further research.

## Conclusions

Using the combined strategy of TMT with LC-MS/MS and subsequent investigation, we confirmed that JWDSD has excellent protective effects in MIRI rats. These protective effects is were associated with Neutrophil extracellular trap formation, RNA polymerase, Serotonergic synapse and Linoleic acid metabolism signaling pathway. Subsequent validation experiments proved that TIAM1 might serve as the targets of JWDSD, that are expected to be developed as candidates against the disease of MIRI in future therapies. These promising results could help to improve the understanding of the effects of drugs on MIRI.

We confirmed that JWDSD has excellent protective effects in MIRI rats using a combined TMT and LC-MS/MS strategy and subsequent investigation. These protective effects were linked to the signaling pathways of neutrophil extracellular trap formation, RNA polymerase, serotonergic synapse, and linoleic acid metabolism. Subsequent validation experiments proved that TIAM1 might serve as the targets of JWDSD, which are expected to be developed as candidates against MIRI disease in future therapies. These promising results could help improve the understanding of the effects of drugs on MIRI.

## Supplementary Information

**Additional file 1.**

**Additional file 2.**

## Data Availability

Not applicable.

## Abbreviations

JWDSD: Jiawei Danshen Decoction
MIRI: Myocardial ischemia-reperfusion injury
TIAM1: T-cell lymphoma invasion and metastasis 1
TMT: Tandem Mass Tag
CK: Creatine kinase
LDH: Lactate dehydrogenase
DEPs: Differentially expressed proteins
KEGG: Kyoto encyclopedia of gene and genomes
GO: Gene ontology.
