# Supplementary material for: TMT-based quantitative proteomics analysis of the effects of Jiawei Danshen decoction myocardial ischemia-reperfusion injury
Source: Proteome Sci. 2022 Dec 14;20:17. doi: 10.1186/s12953-022-00200-7 (PMC9749149; doi:10.1186/s12953-022-00200-7)
Supplement: Supplementary file 1 — Additional file 1. [file 12953_2022_200_MOESM1_ESM.pdf]

湖南中医药大学动物实验福利伦理审查申请表

申请时间：	2020 年 12 月 3 日 星期四			
批准编号：LL2020122401				
一、申请者基本情况				
项目名称	基于 ABHD11-AS1/miR-133a-3p/DAPK2 研究加味丹参饮抗心肌缺血再灌注损伤的机制			
任务来源	湖南省自然科学基金	资助类别	☑1. 纵向 ☐2. 横向	
资助等级	☐1. 国家级 ☑2. 省部级 ☐3. 厅局级 ☐4. 其他			
实验种类	☑1. 医学研究 ☐2. 药物疫苗类 ☐3. 生物类 ☐4. 农业研究 ☐5. 健康食品 ☐6. 其他			
申请单位	湖南中医药大学 药学院			
项目负责人	童巧珍	电话：13975802060	E-mail:qztong88@126.com	
联系人	朱香梅	电话：15874934392	E-mail:2785384490@qq.com	
申请目的	☑初次申请 ☐延长 ☐修改原申请（原批准号： ）			
合作单位				
合作者				
二、所需实验动物				
品种：SD 大鼠	级别：SPF	年龄：		
体重：220-250	雌（只）：0	雄（只）：200		
来源	☑1. 实验动物中心统一采购：<br>☐2. 国内其他正规饲养繁殖单位：<br>☐3. 国外引进：			
饲养场地	☑实验动物中心 ☐自己实验室 ☐其他			

三、以非专业语言简述本研究的目的及对人类、动物或科学的贡献

本实验研究旨在阐明加味丹参饮通过 ABHD11-AS1/miR-133a-3p/DAPK2 抑制 MIR 损伤导致的炎症和自噬，改善 MIR 损伤的机制。通过已知的病理来观察药物的作用机制和效果，为解决实际问题提过理论依据，并且对以后药物的开发提供理论依据。为 IRI 的病理特点和临床治疗研究提供一个可更接近临床、重现性好、塑性好、可控性佳的动物模型。

1、具体说明所选物质的给药方案

药名	给药剂量和频率	给药途径	给药部位	备注
加味丹参饮	$10ml.Kg^{-1}.d^{-1}$	灌胃	口	
单硝酸异山梨酯分散片	$10ml.Kg^{-1}.d^{-1}$	灌胃	口	
生理盐水	1次/d，连续7d	灌胃	口	

2、说明动物保定的必要性，动物保定的方法，包括设备和药物。

抓取大鼠前戴上防护手套，右手轻轻抓住大鼠尾巴的中部并提起，迅速放在笼盖上或其他粗糙面上，左手顺势按、卡在大鼠躯干背部，稍加压力向头颈部滑行，以左手拇指和食指捏住大鼠两耳后部的头颈皮肤，其余三指和手掌握住大鼠背部皮肤，完成抓取保定。麻醉的大鼠可置于大鼠实验板上（仰卧位），用橡皮筋固定好四肢（也可用棉线），为防止咬伤人，应用棉线将大鼠两上门齿固定于实验板上。

3、标本采集方案

采集的组织或液体	采集方法	数量或体积	采集频率	持续时间或最大采集数量
心肌组织	手术 开胸	200	半个月	采集过程一般5分钟
动物采血	麻醉、腹腔动脉抽血	200	半个月	采集过程一般5分钟

4、动物标识

☑染色　　☐耳标　　☐芯片

是否在同一动物上进行多个操作	☐ 否<br>☑是，具体说明：需要在动物身上手术，并灌胃给药

七．导致疼痛的分类

☐A. 无疼痛

☐B. 一般性疼痛

☐C. 轻微疼痛

☑D. 有疼痛，但能够解除

☐E. 不能缓解的疼痛

八．麻醉、镇痛

药物名称	给药剂量和频率	给药途径	维持时间
水合氯醛	10%浓度，3.5ml/1Kg	腹腔注射	1-2 个小时

九．人道主义结束动物生命

A、安乐死

☐ 1、迅速断头

☑ 2、头颈部迅速脱臼 （<1kg）

☐ 3、在全身麻醉下放血（适合猫，反刍动物，马，猪等 ）

☐ 4、过量吸入麻醉剂（氟烷、异氟醚、甲氧氟烷等）

☐ 5、腹腔注射安乐死药剂

☐ 6、静脉注射安乐死药剂

☐ 7、二氧化碳或二氧化碳/氧气混合气体

☐ 8、其它 ，请具体说明

B、剩余动物的最终处理

☑1、对动物实施安乐死

☐2、返回生产/育种单位

☐3、作其它研究

☐4、动物饲养在动物中心，直到其自然死亡

☐5、其它，具体说明：

C、动物尸体、组织、或体液的最终处理

☐1、制作标本

☑2、袋装后冷冻，由学校实验动物中心作无公害化处理

☐3、其它 ，具体说明

十、有害（毒）物质的使用（应得到安全委员会的批准）

	是	否	使用物品的名称
1 放射性同位素	☐	☑	
2 生物物品	☐	☑	
3 有毒化学品、药品	☐	☑	
4 重组 DNA	☐	☑	
5 其它	☐	☑	

十一、实验动物设施准入资格：

实验动物从业人员	设施准入资格证号码
朱香梅	202007067

十二、承诺

项目负责人承诺书

我承诺该申请使用表的内容准确无误。

我同意遵守中华人民共和国国家科学技术委员会制定的《实验动物管理条例》、中华人民共和国科学技术部发布的《关于善待实验动物的指导性意见》、湖南省人民政府发布的《湖南省实验动物管理办法》。

我承诺包括我自己在内的该申请使用表中提及的与实验动物有接触的人员，已经参加了湖南中医药大学实验动物中心要求的相关培训，掌握了申请使用表中涉及的动物实验方法，都有能力完成动物实验，并且深知使用这些活体动物及动物组织所存在的风险。

我清楚作为该项目的负责人，有责任承诺本课题组所有成员在本研究工作中均会遵循人道主义原则，确保实验动物的福利伦理，并严格遵守湖南中医药大学实验动物中心的相关规章制度。

项目负责人签字：[illegible]　　动物实验负责人签字：林香梅

日期：2020年 12 月 24 日

十三、审核意见：

通过

主审委员签字：

日期：2020年 12 月 24 日

湖南中医药大学实验动物伦理委员会
