# Supplementary figures and images for: TMT-based quantitative proteomics analysis of the effects of Jiawei Danshen decoction myocardial ischemia-reperfusion injury
Source: Proteome Sci. 2022 Dec 14;20:17. doi: 10.1186/s12953-022-00200-7 (PMC9749149; doi:10.1186/s12953-022-00200-7)

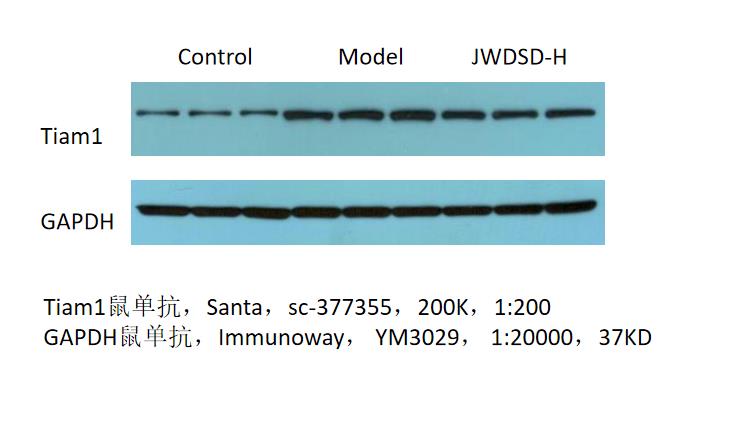

Supplement: Supplementary file 2 — Additional file 2. [file 12953_2022_200_MOESM2_ESM.jpg]
